# Elimination of lymphatic filariasis as a public health problem in Niue under PacELF, 1999–2016

**DOI:** 10.1186/s41182-019-0141-1

**Published:** 2019-03-15

**Authors:** Catherine N. Carlingford, Wayne Melrose, Grizelda Mokoia, Patricia M. Graves, Kazuyo Ichimori, Corinne Capuano, Sung Hye Kim, Padmasiri Aratchige, Manila Nosa

**Affiliations:** 1 School of Public Health, The University of Sydney, Camperdown, Australia; 2 College of Public Health, Medical and Veterinary Sciences, James Cook University, Townsville and Cairns, Australia; 3 Niue Health Department, Alofi, Niue; 4 Institute of Tropical Medicine, Nagasaki University, Nagasaki, Japan; 5 WHO Office of the Representative for the South Pacific and Division of Pacific Technical Support, Suva, Fiji; 6 Department of Parasitology, Liverpool School of Tropical Medicine, Liverpool, UK; 7 Niue Health Department, Alofi, Niue

**Keywords:** Lymphatic filariasis, Mass drug administration, Niue, Microfilaria, Epidemiology, *Wuchereria bancrofti*, PacELF

## Abstract

**Background:**

Lymphatic filariasis (LF) is a mosquito-borne parasitic disease which is targeted for elimination as a public health problem worldwide. Niue is a small self-governing South Pacific island nation with approximately 1600 residents that was formerly LF endemic. Here, we review the progress made towards eliminating LF in Niue since 1999.

**Methods:**

This study has reviewed all the available literature relating to LF in Niue to assess surveillance efforts and the elimination of transmission. Reviewed documentation included both published and unpublished works including historical reports of LF, WHO PacELF records, and Niue Country Reports of the national LF elimination program.

**Findings:**

Niue conducted mapping of baseline LF endemicity by testing the total present and consenting population for LF antigen with immunochromatographic test (ICT) in 1999, when circulating filarial antigen prevalence was 3.1% (n = 1794). Five nationwide annual mass drug administration (MDA) rounds with albendazole (400 mg) and diethylcarbamazine citrate (DEC) were undertaken from 2000 to 2004, with coverage reported from distribution records ranging from 78 to 99% of the eligible population, which excluded pregnant women and children under 2 years of age. A further whole population survey using ICT in 2001 found 1.3% positive (n = 1630). In 2004, antigen prevalence had reduced to 0.2% (n = 1285). A similar post-MDA survey in 2009 indicated antigen prevalence to be 0.5% (n = 1378). Seven positive cases were re-tested and re-treated every six months until negative.

**Conclusions:**

After five rounds of MDA, Niue had reduced the LF antigen population prevalence in all ages from 3.1% to below 1% and maintained this prevalence for a further  five years. Due to Niue’s small population, surveillance was done by whole population surveys. Niue’s results support the WHO recommended strategy that five to six rounds of annual MDA with effective population coverage can successfully interrupt the transmission of LF. Niue received official acknowledgement of the validation of elimination of LF as a public health problem by the WHO Director-General and WHO Western Pacific Regional Office (WPRO) Regional Director at the 67th session of the Regional Committee for the Western Pacific held in Manila in October 2016.

## Background

Lymphatic filariasis (LF) is a mosquito-borne parasitic disease. Worldwide, the disease is caused by three species of parasitic worm, namely *Wuchereria bancrofti*, *Brugia malayi*, and *Brugia timori* [1]. The parasites are transmitted to humans via mosquitoes from the *Anopheles*, *Aedes*, *Culex*, and *Mansonia* genera [2]. In the South Pacific, *W. bancrofti* is the parasite responsible for LF [1]. Parasites enter the body after a blood meal and settle in the lymphatic system where they mature, mate, and release millions of microfilariae into the bloodstream. Early stages of infection are frequently asymptomatic [1], although intermittent episodes of acute dermatolymphangioadenitis and acute filarial lymphangitis occur [3, 4]. If untreated infection persists, secondary bacterial infections and inflammation occur, and damage caused to the lymphatic system accumulates, resulting in some individuals in severely disabling and disfiguring manifestations of lymphoedema, elephantiasis, and hydrocele [1, 4]. Globally, LF is the second leading cause of chronic disability [5]. The disease imposes considerable economic and psychosocial hardship on sufferers, their carers, and families [6–8].

In 1997, the Member States of the WHO at the World Health Assembly (WHA) committed to eliminating LF as a public health problem internationally through Resolution WHA50.29 [9]. As a result, the World Health Organization (WHO) launched the Global Programme to Eliminate Lymphatic Filariasis (GPELF), with the aim of eliminating LF by the year 2020 [5]. The strategy included interrupting the transmission of LF through mass drug administration (MDA) and alleviating current suffering through improved morbidity management and disability prevention (MMDP) [5, 10, 11]. In 1999, under the auspices of the WHO, the regional arm of GPELF, the Pacific Programme to Eliminate Lymphatic Filariasis (PacELF), commenced in 22 Pacific Island Countries and Territories (PICTs) [1, 12, 13] and included endemic island nations such as Niue.

**Fig. 1:**
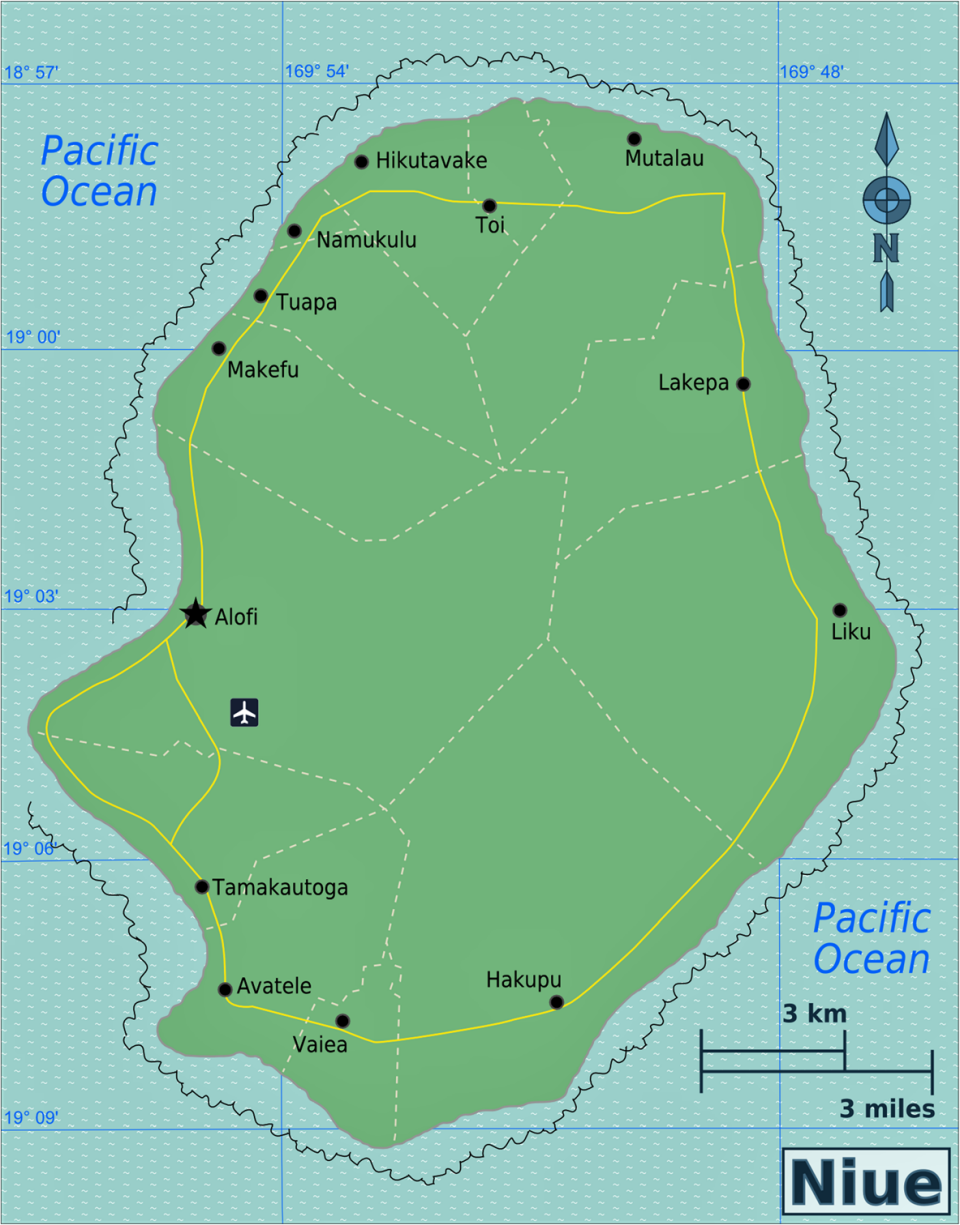
Niue and 14 districts (villages). Source [28]

Niue (*NEW-ay*) is a self-governing South Pacific Ocean island. The elevated coral atoll is located approximately 660 km southeast of Samoa, 480 km east of Tonga, and 2400 km northeast of New Zealand (19° S 169° W) [14]. The island covers approximately 261.46 km^2^ and is divided into 14 administrative areas, with the capital, Alofi, divided into two districts: Alofi South and Alofi North (Fig. 1).

Niue’s population has been in decline for many decades. In 1971, Niue’s population was 4990. Twenty-six years later in 1997, however, the population had fallen to 2088 residents [14]. Emigration loss to neighboring New Zealand (NZ) has been cited as a contributing factor to population decline [15]. Figures suggest roughly 90% of Niue’s population live and work in NZ where they concurrently hold automatic citizenship [14]. NZ census data (1996) indicated approximately 18,477 Niueans resided in NZ [16]. More recent estimates suggest Niue’s population is roughly 1611 (2011), with population diversity broken down into Niuean (66.5%), part-Niuean (13.4%) (Tongan, Tuvaluan, Samoan), and non-Niuean (20.1%) (Asian and European) [1, 14]. Niue residents enjoy a relatively high standard of living. All people have universal access to free education and healthcare; housing and hygiene are all of relatively high standard [17]. Niue’s climate is tropical. Average daily temperatures range from 24 to 27 °C [14]. The wet season from November to April brings high humidity, rainfall, and warm temperatures which are favorable conditions for mosquito breeding [14]. The primary mosquito vector responsible for transmitting LF throughout Polynesia is the genus *Aedes* (*Ae.*) [18]. In Niue, the specific species responsible for transmitting LF is *Ae. cooki* [1]. *Ae. cooki* bites during the day, dusk, and dawn and commonly breeds in stagnant water containers such as cisterns and coconut shells [15].

### Monitoring and surveillance frameworks: GPELF and PacELF guidelines

The Global Programme to Eliminate Lymphatic Filariasis (GPELF) recommended that elimination efforts occur in programmatic stages. These included mapping, MDA, sentinel site surveillance during MDA, “stop MDA decision” surveys, post-MDA surveillance, and verification [11]. Mapping required countries to survey and map LF to determine whether Mf or antigenemia (Ag) prevalence was > 1%, in which case countries were considered endemic [11]. Endemic countries required annual MDAs be undertaken for at least five rounds, consisting of combined albendazole (400 mg) plus diethylcarbamazine citrate (DEC) (6 mg/kg) or (in countries where onchocerciasis is co-endemic) ivermectin (150–200 μg/kg) [6, 11]. Sentinel sites (villages surveyed longitudinally) and spot check village surveys were done during MDA and prior to larger surveys to decide whether MDA could be stopped. After MDA was stopped, post-MDA surveillance was required to monitor infection levels for approximately five years.

The initial recommendations for stopping MDA surveys were for lot quality assurance sampling (LQAS) surveys in 3000 children aged five years, to determine whether transmission was still occurring, with an elimination criterion of < 0.1% circulating filariasis antigen (CFA) prevalence [1, 19]. The rationale for surveying children was that if adequate MDA coverage (> 65% of the eligible population) had decreased population infection rates, this would prevent transmission and thus infection in young children who were born after the commencement of the MDA [20]. However, the PacELF program did not feel confident in using only young children for the stop MDA decision, especially in small countries with few children. Therefore, a survey of all age groups through community cluster sampling (known as the PacELF C-survey) was used to decide whether to stop MDA [1].

Revised GPELF guidelines in 2011 modified the LQAS method, and the cutoffs for “passing” a transmission assessment survey (TAS) to < 1% CFA prevalence in five-to-six-year-olds (in *Aedes* transmission areas) [11, 20]. The TAS was recommended by GPELF to be used for both the stop MDA decision and for post-MDA surveillance of at least two more TAS surveys at two-to-three-year intervals [6]. This is in contrast to earlier PacELF guidelines mentioned above, in which the recommendation was to survey all ages for the stop MDA decision and ensure antigen prevalence in all ages was < 1%. Under PacELF, once MDA was stopped, surveys in children only would then be done, as for TAS.

**Fig. 2:**
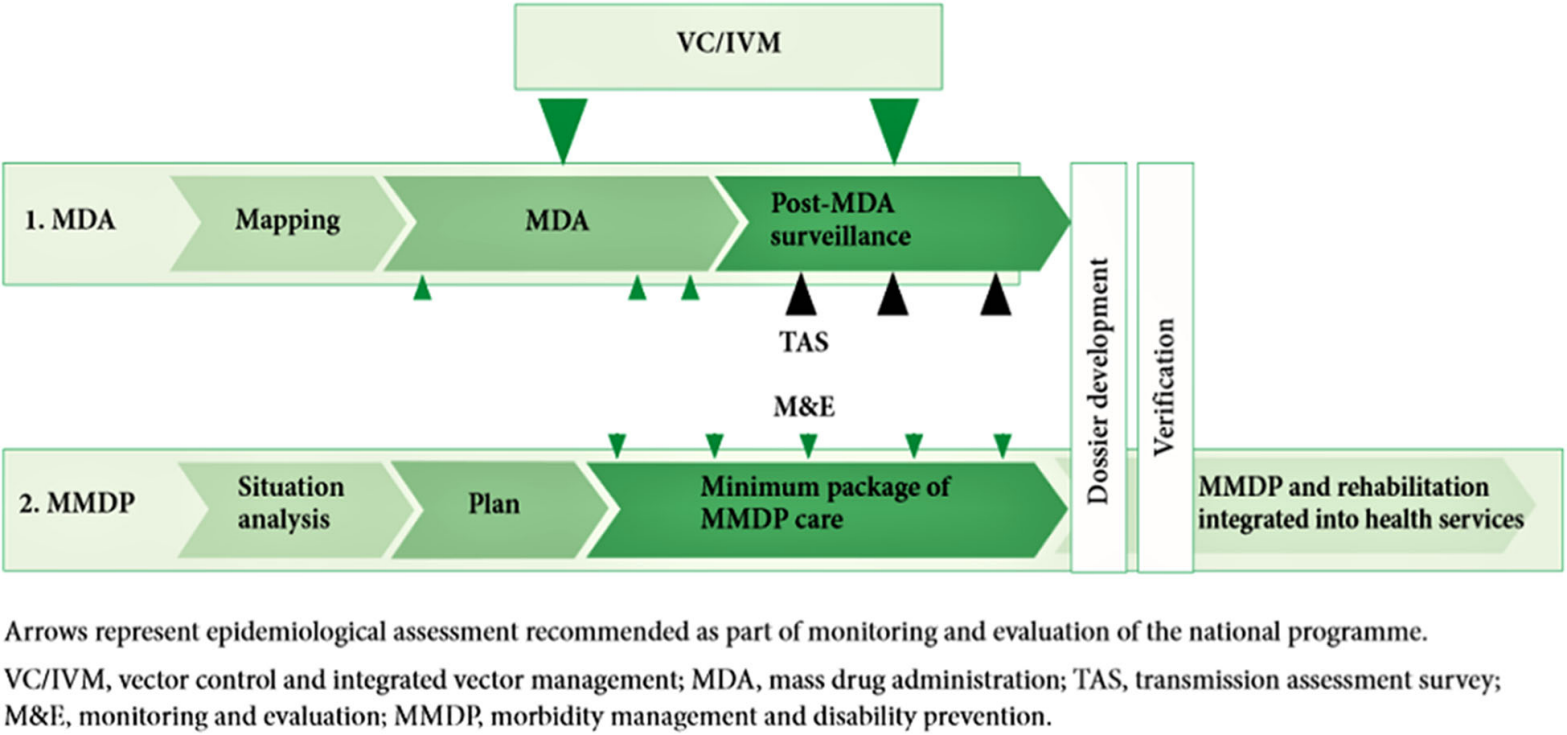
The Global Programme to Eliminate Lymphatic Filariasis (GPELF) Strategy. Arrows represent epidemiological assessment recommended as part of monitoring and evaluation of the national program. VC/IVM, vector control and integrated vector management; MDA, mass drug administration; TAS, transmission assessment survey; M&E, monitoring and evaluation; MMDP, morbidity management and disability prevention. Source [20]

For validation of elimination (assuming all TAS were passed and sufficient time elapsed since MDA), countries then develop a dossier detailing national epidemiological data and evidence of the absence of transmission of LF to support their claim. This dossier is reviewed by an independent Dossier Review Group and the WHO Regional Programme Review Group. If successful, the country receives an official acknowledgement by the WHO as having successfully eliminated LF as a public health problem [6]. This elimination process is illustrated in Fig. 2.

Unlike many GPELF countries, the smaller population sizes of many PICTs made the initial LQAS low-level elimination target (0.1%) difficult. In response, the WHO Western Pacific Regional Office (WPRO) program (PacELF) developed its own monitoring and evaluation framework. In 2003 and 2004, PacELF guidelines used a community cluster survey in all ages for the decision to stop MDA, with an elimination target of antigen prevalence < 1% where *W. bancrofti* was endemic and *Aedes* the principal vector [11].

Niue was a special case within PacELF. The program spanned the time period of both PacELF and GPELF guidelines, but in fact, due to Niue’s small population size, the program conducted only whole population surveys rather than sentinel sites, village cluster surveys, or TAS/child transmission surveys at all program decision points.

### History of LF in Niue prior to PacELF

Suspect cases of LF existed in the Pacific Islands as early as in 1785 when Captain James Cook noted symptoms in Tongans that closely resembled elephantiasis [1]. In 1954, a microfilariae (Mf) survey was undertaken in Niue to assess the number of positive carriers, revealing a prevalence of 22.2% (166/748) [17, 21]. Three MDAs were undertaken before the commencement of PacELF. The first MDA was in 1956, the second in 1972, and the third in 1997 [1]. There were four large blood surveys conducted between 1956 and 1996, although the sampling methodology used and age groups tested are not clear.

The initial MDA in January of 1956 consisted of DEC 50 mg administered monthly [17]. Reports of pain in the joints and limbs were noted in asymptomatic positive carriers 24 h following MDA [17]. A follow-up survey conducted in December of 1956 on the population aged six years and older showed Mf prevalence had reduced to 3.0% (83/2791) [17]. Positive individuals were treated intensively with DEC 50 mg three times daily for two weeks with a week interval between another week of treatment, continuing until individuals tested Mf negative. Four years later, in 1960, another Mf survey revealed population prevalence had risen slightly to 3.2% (31/957) [22].

Sixteen years after the first 1956 MDA and twelve years after the 1960 survey, a Mf survey in 1972 of almost the whole population (99.7%) revealed that prevalence had risen to 16.4% (724/4408) [23]. Subsequently, in 1972, a second MDA was carried out with DEC (6 mg/kg once weekly for twelve weeks, followed by once monthly for twelve months) [23]. Coverage was reported as 98.4% although the denominator used (total or eligible population) was not clear, nor whether this was for the entire population or a sample of the entire population. However, only 56.6% of the targeted population completed the full course of MDA and no follow-up surveys were undertaken [15].

**Fig. 3:**
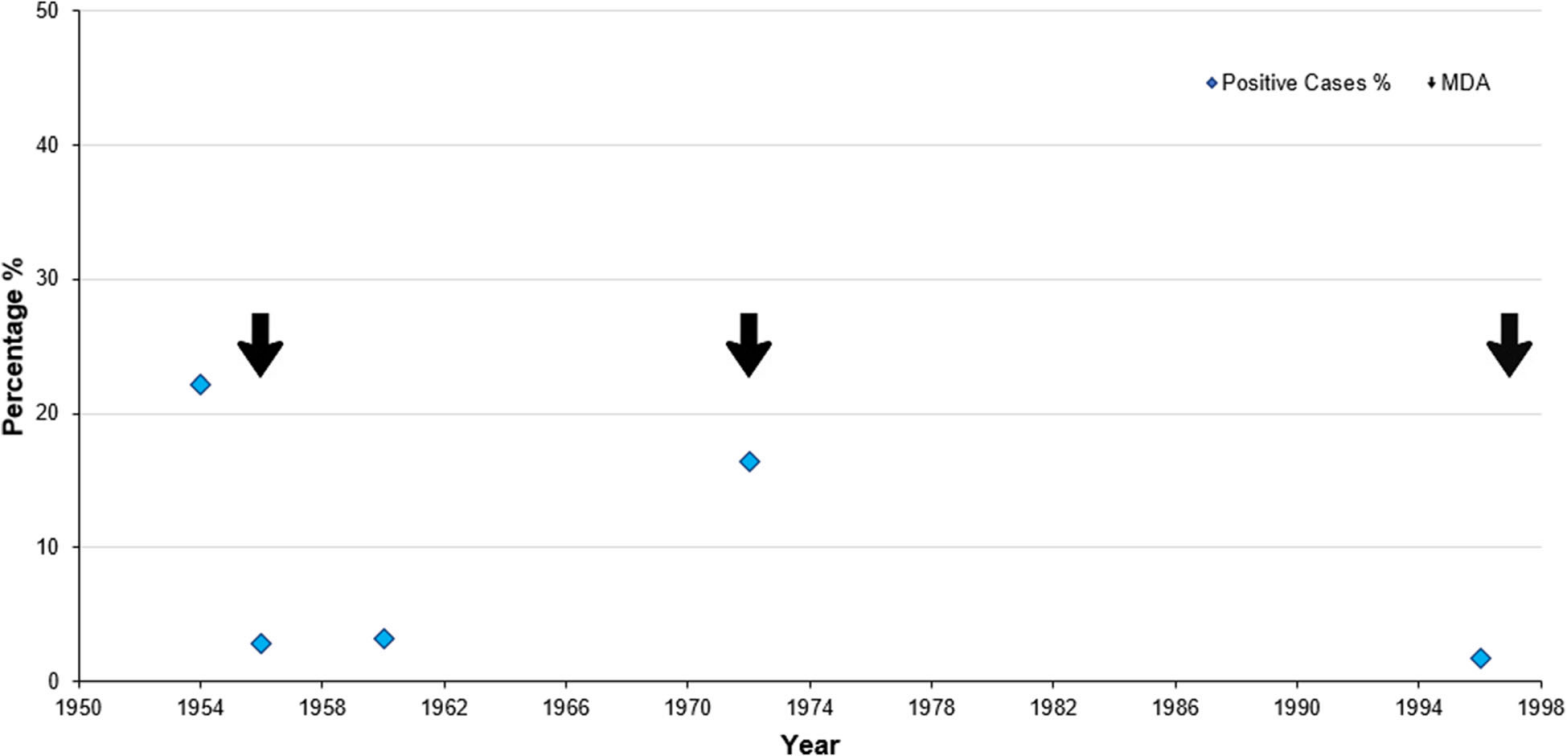
LF monitoring and surveillance history prior to PacELF in Niue (1950–1998). *Y*-axis, percentage %; *X*-axis, year

In 1996, a survey on 82% of the population revealed a Mf prevalence of 1.8% (26/1471) [24]. In 1997, a third whole-population MDA was undertaken (on those 4 years or older) with combined DEC (6 mg/kg) and ivermectin (200 μg/kg), with a reported coverage of 87% [15]. These surveillance and control activities are displayed in Fig. 3.

## Methods

During October of 2017, we collected and reviewed both unpublished and published WHO and PacELF program data relating to Niue. We also searched for associated literature regarding LF elimination and surveillance and control activities in Niue. Types of literature retrieved included PacELF data books including MDA data and blood survey results, program guidelines, unpublished data, and country reports. Other papers we reviewed were historical filariasis reports relating to Niue and more recently published peer-reviewed literature. Where literature was not accessed through the WHO Collaborating Centre PacELF data warehouse held at James Cook University, it was available freely online. We have reviewed this literature and extracted key data to write and inform this review.

### Findings

PacELF was launched in 1999. In September of the same year, an initial baseline survey of LF antigenemia prevalence was undertaken on Niue’s whole population (aged two years and older). Niue’s population antigen prevalence was 3.1% [22] and, therefore, required five rounds of MDA. All antigen prevalence testing was undertaken using a standardized immunochromatographic rapid card test (ICT) which has high sensitivity (96–100%) and specificity (95–100%) [8].

Prior to the first MDA, a national awareness campaign was undertaken, including a television awareness program, a radio announcement, and a national distribution of filariasis leaflets [15]. In 2000, the first national MDA round was undertaken using a combined regimen of DEC (6 mg/kg) and albendazole (400 mg). The main distribution method was people coming to the village community halls to obtain medicines from trained distributors who insisted upon directly observed therapy (DOT) [15]. Absentees had to come and pick their medicines at the hospital or it was delivered to their homes following the MDA. The second nationwide MDA round was undertaken in March of 2001 using the aforementioned drug regimen. In September of the same year, a whole-population survey was undertaken by ICT. In March of 2002 and 2003, third and fourth national MDA rounds were undertaken (using the aforementioned drug regimen), and in addition, the previously positive cases were followed up and treated if necessary. In July of 2004, a fifth and final MDA round was undertaken and was followed up by a national stop MDA C-survey in August. Five years after the last MDA round, in 2009, a whole-population survey was again undertaken. Those who tested positive were re-tested and re-treated every six months until the results became negative. In total, five annual rounds of MDA were completed. A timeline showing these programmatic and surveillance activities is given in Table 1.

**Table 1:** Program timeline and surveillance activities in Niue, 1999–2017

Year	Activity	Detail and location of activity
1999	Baseline survey	Whole population mapping baseline survey (by ICT)
		Nationwide education and awareness campaign
2000	MDA1	Nationwide MDA (combined DEC and albendazole)
2001	MDA2	Nationwide MDA (combined DEC and albendazole)
	Mid-term survey	Mid-term whole population survey (ICT)
2002	MDA3	Nationwide MDA (combined DEC and albendazole)
	Follow-up	Testing (by ICT) of positive cases and treatment
2003	MDA4	Nationwide MDA (combined DEC and albendazole)
	Follow-up	Testing (by ICT) of positive cases and treatment
2004	MDA5	Final Nationwide MDA (combined DEC and albendazole)
	Stop MDA survey	Nationwide Survey by ICT in all ages
2006	Follow-up	Follow-up of one +ve case undertaken
2009	Post MDA survey	Nationwide Post-MDA survey (by ICT all ages)
	Follow-up	+ve cases re-tested, re-treated every six months until negative
2012	Dossier development	Dossier for the validation of elimination of LF prepared
2013	Dossier submission	Validation dossier submitted to WHO Review Group
2016	Elimination validation	Official acknowledgement received from WHO of the validation of elimination of LF as a public health problem

**Fig. 4:**
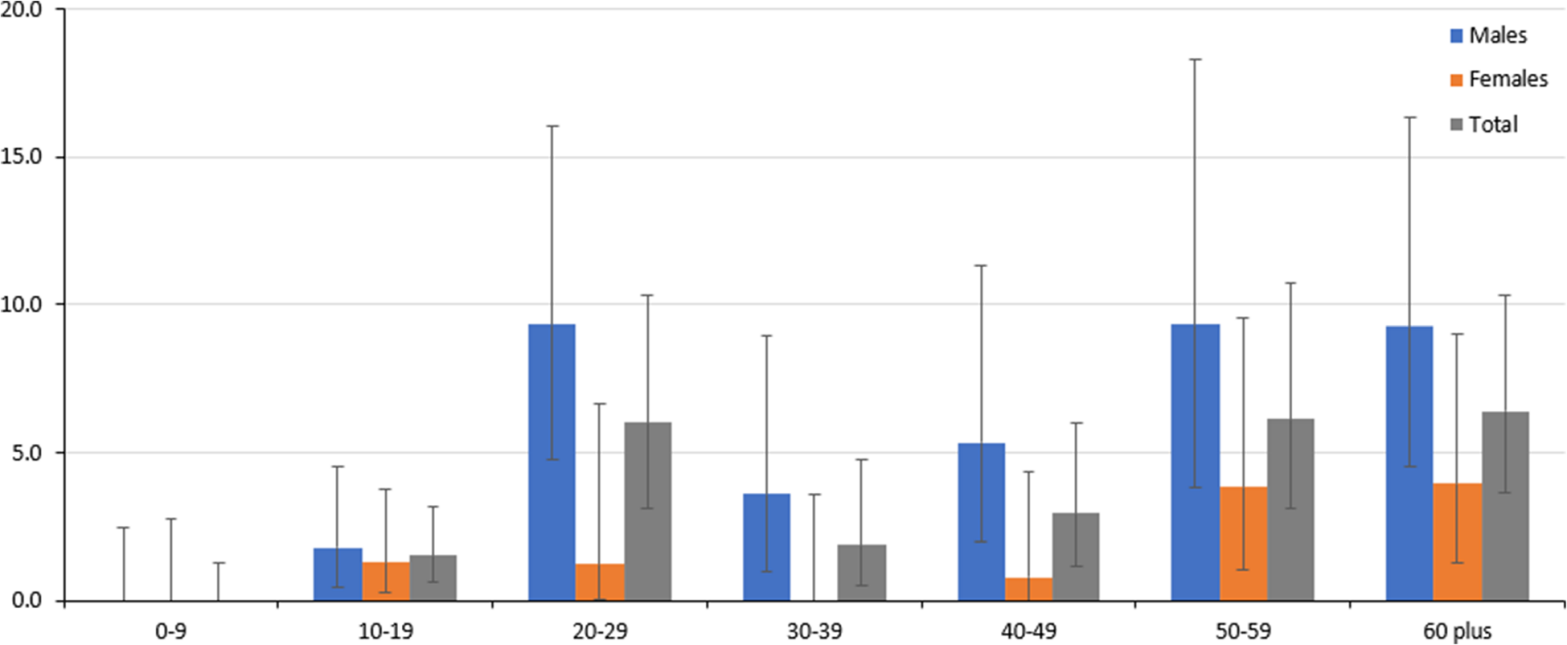
Percentage of positive cases (ICT) by age and gender, Niue 1999. *Y*-axis, percentage positive (95% confidence interval); *X*-axis, age group. Source: Adapted from [22]. Error bars represent binomial 95% confidence intervals

In 1999, Niue’s baseline survey revealed an antigenemia prevalence of 3.1% (56/1794) M = 42, F = 14 [22]. High numbers of positive cases were seen in Hikutavake (*n* = 13) and Tamakautonga (*n* = 13) villages, located north and southwest of the island, respectively [22]. These are coastal villages (Fig. 1) with adjacent rural areas that can harbor mosquitoes, unlike other villages on the island from Vaiea to Mutulau. No children (< ten years old) at baseline tested positive (*n* = 280); however, there were seven positives in the 10–19 years age group (*n* = 453). Prevalence was highest in those aged 20–29, 50–59, or 60+ years with consistently more positive results recorded for males than females (Fig. 4).

After MDA2 in 2001, a whole population survey showed antigenemia prevalence had fallen to 1.3% (22/1630) with positive cases seen in, amongst other locations, Tamakautonga village (*n* = 4) and Alofi North (*n* = 4) [22]. In 2002, a follow-up survey of positive cases that had been identified in previous surveys (*n* = 20) revealed 12 tested ICT positive. A further follow-up survey in 2003 of positive cases (*n* = 26) revealed 16 tested ICT positive [22]. All cases were treated.

After MDA5 in 2004, a whole-population stop MDA survey revealed antigenemia prevalence to be 0.2% (3/1285) [18]. No further MDA was given after 2004. A whole-population survey in 2009 found an overall antigenemia prevalence of 0.51% (*n* = 1378) and no positive cases in six to seven year-old children. The seven individuals who tested positive in 2009 were re-tested with blood ICT tests and treated (if positive) every six months until testing negative (Table 2).

**Table 2:** Results of filarial blood surveys in Niue under PacELF

Year	Sample	ICT examined (*n*)	ICT positive (*n*)	ICT positive (%)
1999	Whole population	1794	56	3.1
2001	Whole population	1630	22	1.3
2002	Follow-up of + ve cases	20	12	60.0
2003	Follow-up of + ve cases	26	16	61.5
2004	Whole population	1285	3	0.2
2006	Follow-up + ve cases	1	0	0.0
2009	Whole population	1378	7	0.5

Source: Adapted from [22, 27]

**Fig. 5:**
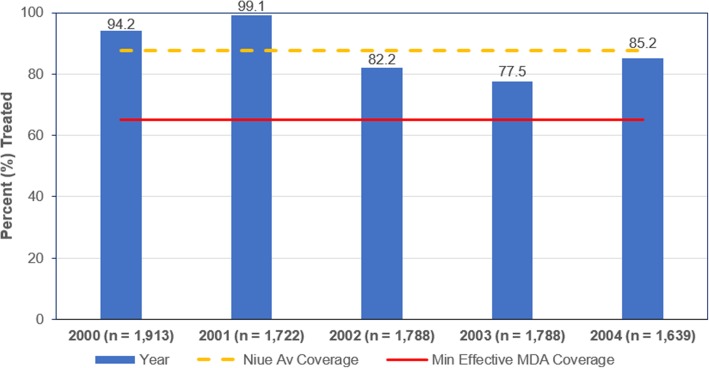
Annual MDA coverage under PacELF Niue 2000–2004. *Y*-axis, percent (%) treated. Source: Adapted from [22, 27]

The minimum coverage for annual MDA to be considered effective was ≥ 65% of the total population [20]. Niue reported high rates of effective coverage based on the census population denominator (Fig. 5). The average MDA coverage across the five MDA rounds was 87.7% (Fig. 5).

Only a small proportion of the population did not participate in MDA. The principal reason reported for not participating in MDA was absence at the time of drug distribution. Absenteeism was the primary reason given for non-treatment in MDA2 (73% of 142 reasons of any type), MDA3 (66% of 105 reasons of any type), and MDA4 (63% of 126 reasons of any type). The main reason for absence was residents who had migrated to NZ and students studying overseas. Some new residents or expatriates did not participate if they were not fully aware of the program. In the second MDA, the highest reported absences were recorded in Lakepa (*n* = 30), followed by Alofi North (*n* = 23). In the third MDA, the highest recorded absences were in Alofi South (*n* = 30) followed by Alofi North (*n* = 19). In the fourth MDA, the highest reported absence was seen in Tuapa (*n* = 39) followed by Alofi North (*n* = 12) [22]. Aside from absenteeism, other reported reasons for not engaging in MDA were pregnancy, infancy, or a small number due to extreme age [22]. There were no formal reports of adverse drug reactions (ADRs) from MDA, but informal complaints of fatigue and sore body were noted. This data could be included amongst reasons for missing MDA listed in data books as “Other”, although this remains undistinguishable [22].

### MMDP: LF morbidity in Niue

For every person with lymphoedema, elephantiasis, or hydrocele, a minimum package of care for management of morbidity and prevention of disability (MMDP) must be made available (Fig. 2) [20, 25]. This care must include MDA or individual treatment to destroy remaining adult parasites and microfilaria, surgery for hydrocele in *W. bancrofti* endemic areas, treatment for episodes of adenolymphangitis (ADL), and management of lymphoedema [6, 25]. In 2012, it was reported that there had been no known cases of elephantiasis or filarial hydrocele in Niue for more than a decade and that the last person suffering from elephantiasis died twelve years ago [15]. Since then, there have been no reports of filarial hydrocele or acute filariasis [15].

## Discussion

Several factors have supported the LF elimination success seen in Niue. Firstly, Niue had a small, easily-accessible resident population, good leadership, and committed Health Department staff as well as good communication with the villages' councils, meaning effective coverage rates were possible throughout the program (> 65%). Niue also had a low initial baseline antigen prevalence of 3.1% (by ICT) [18]. The national LF elimination program in Niue also actively pursued individuals who were absent at the time of each MDA to ensure future treatment (e.g., residents returning from overseas who were prompted to collect their tablets). This also contributed to Niue being the only PacELF program to undertake whole-population surveys with reliable cross-sectional data, rather than undertaking stratified cluster surveys or child transmission surveys like other LF programs [18].

There are some limitations to this study. Information on LF prior to 1999 does not report the sampling method used to test individuals or the population denominator for studies. The population of Niue fluctuates widely, with a large proportion of the population traveling temporarily or permanently to NZ. The population has dropped from around 5000 in the 1960s to less than 2000 today. The number tested in surveys during PacELF represents an unknown (although large) proportion of the resident population. It is not clear why there was an increase in the number of positive and examined cases in 2003, compared with the previous 2002 follow-up survey.

A factor that may have supported success for the LF program was that, prior to the MDA period with the PacELF program, Niue had a de-worming program targeting school-aged children for over ten years. This may have contributed to the finding that no children with positive ICT were found pre- or post-MDA. As there is only one primary school in Niue, it was easy for a nurse to monitor and ensure children completed treatment. There was a stool survey undertaken by WHO in 2002 in which no helminths were found in local children, except one which was an imported case.

During the pre-MDA period, there was limited movement of people in and out of Niue, as there was only one flight to and from the island each week. The number of absentees tended to increase when more students left the island for studies overseas, and some residents left for medical treatment in Auckland after which they needed clearance to return to the island. The number of immigrants during the pre-MDA period was also restricted due to a government immigration policy: a health clearance was required before an entry permit was granted only for those who wanted to set up permanent residence on the island. During the MDA program (which was also occurring in other Pacific countries and territories), permanent residents from endemic countries such as Tonga, Samoa, or Tuvalu who wished to migrate to Niue but were not tested or treated in their home country were offered ICT. However, no positives were found. From 2012, as part of immigration requirements, any external applicants seeking residency (from another Pacific island) had to undergo an LF ICT test and, if found positive, were offered treatment.

Niue’s success may have been further augmented by the island’s natural climatic variability. Dry seasons in Niue reduce the vector’s breeding sites, limiting transmission to primarily the wet season rather than year-round [15]. This, in addition to Niue’s consistent vector control efforts (village inspections, actively destroying man-made breeding sites) to concurrent control for dengue [15], could have contributed to the elimination success seen in Niue. Further promotors of Niue’s success include a well-conducted program, strengthened by a constant program manager for over ten years [15]. It is widely acknowledged that skilled workforce migration and “brain drain” are common in low-resource settings [26]. Stable leadership throughout the national LF elimination program in Niue has, therefore, likely influenced its success. Similarly, Niue’s program received strong political support from the Minister of Health and sound technical advice from the WHO to the program manager [15]. Political commitment and will are essential to good health governance and expanding responsibilities for health [26]. Lastly, unlike prior efforts to eliminate LF in Niue, the MDA rounds in PacELF consisted of a proven combination of DEC and albendazole and continued for five consecutive rounds until minimum thresholds were reached. Unlike in the past, the MDA rounds were stopped only after assessing that the PacELF elimination threshold level of 1% in all ages was reached by testing the whole population. This was also enhanced by the use of DOT in Niue. Wider public–private partnerships between WHO, GlaxoSmithKline (GSK), and the Japan International Cooperation Agency (JICA) enabled mass donations of DEC (105,000 tablets), albendazole (7400 tablets), and 6000 ICT testing kits to be made available to Niue and contribute to the overarching program’s success [1, 5].

## Conclusion

At the commencement of PacELF in 1999, Niue’s national CFA prevalence in all ages was estimated at 3.1%. After five rounds of MDA with combined DEC and albendazole, this had reduced in all ages to 0.2% in 2004. A post-MDA survey in 2009 showed a remaining antigen prevalence of 0.51% with no positive cases in children aged six to seven years, suggesting transmission has ceased and no recrudescence had occurred after the stoppage of MDA. Results in Niue support the WHO recommendation that five to six rounds of MDA with an effective population coverage can successfully interrupt LF transmission. In 2012, the Niue Health Department compiled a dossier for its application for the validation of the elimination of LF as a public health problem. The dossier summarized surveillance and control activities undertaken throughout the program. The dossier was submitted to WHO in 2013 and was reviewed during the meeting of the Western Pacific Regional Programme Review Group (RPRG) in July 2014. An official acknowledgement of the elimination of LF as a public health problem was given to Niue by the WHO Director-General and the WHO WPRO Regional Director during the 67th session of the Regional Committee held in Manila in October 2016. It should be noted that LF is not eliminated globally. The status of LF elimination as a public health problem is also not irreversible; therefore, Niue will need to remain vigilant in detecting any recrudescence of the disease.

## Abbreviations

Ag: Antigenaemia
DEC: Diethylcarbamazine citrate
GPELF: Global Programme to Eliminate Lymphatic Filariasis
ICT: Immunochromatographic test
LF: Lymphatic filariasis
MDA: Mass drug administration
Mf: Microfilariae
PacELF: Pacific Programme to Eliminate Lymphatic Filariasis
TAS: Transmission assessment survey
WHA: World Health Assembly
WHO: World Health Organization
WPRO: WHO Western Pacific Regional Office
